# Bioactive Salen-type Schiff Base Transition Metal Complexes as Possible Anticancer Agents

**DOI:** 10.22037/ijpr.2019.12792.11151

**Published:** 2019

**Authors:** Maryam Damercheli, Mahdi Mahdi, Bita Mehravi, Mehdi Shafiee Ardestani

**Affiliations:** a *Department of Inorganic Chemistry, Faculty of Chemistry, Semnan University, Semnan, Iran. *; b *Department of Medical Nanotechnology, Faculty of Advanced Technology in Medicine, Iran University of Medical Sciences, Tehran, Iran. *; c *Department of Radiopharmacy, Faculty of Pharmacy, Tehran University of Medical Sciences, Tehran, Iran.*

**Keywords:** Histone deacetylase, Anticancer, Schiff base, Docking

## Abstract

Although metal-based anticancer drugs have been recognized as the most effective agents over the organic compounds, non-selectivity and high toxic effects have limited their applications in a way that only three Pt-analogues have progressed into clinical use. These problems have spurred chemists to develop different strategies based on alternative targets. This work focuses on predicting potency and mode of interactions of a series of salen type Schiff base transition metal complexes derived from meso-1,2-diphenyl-1,2-ethylenediamine, over some proteins (HDAC7, HDAC2, CatB, B-RAF kinase, TopII, RNR, TS, and rHA) using computational docking method, to be later considered as possible anticancer agents. The obtained results showed that all complexes exhibited higher affinity for HDAC7 than the other targets. Moreover, the bromo-derivatives of the copper compounds were more active on HDAC7 than the other derivatives. Such bromo compounds showed considerable interactions with Kinase, RNR, TS, and CatB. Contrary to Histone deacetylase (HAD)C7; HDAC2 was predicted to be relatively poor target. As expected, formation of the hydrophobic interactions between the metal complexes and the protein targets were essential for activity of the metal compounds. This study provides some more information for further optimizations and development of new metallodrugs as enzyme inhibitors for potential therapeutic agents.

## Introduction

Since the outstanding discovery of the anticancer properties of cis-platin, and because of the wide variety of available reactivity, inorganic compounds have received noticeable attention in biological chemistry (1-4). Traditionally, DNA is the main target of the metal-based anticancer drugs (5). Therefore, besides acting on tumor tissues, other healthy organs are also affected, causing severe undesirable side effects. The lack of selectivity is a potential hinder of the aforementioned metal complexes. To date, several new anticancer targets are being discovered due to increase available information in molecular biology of the cancer cells. Among the targets identified, kinases (6, 7), histone deacetylases (8, 9), proteases (10, 11), topoisomerases (12, 13), and a number of other protein targets (14-16) have been broadly studied as biomolecular targets for metal-based drugs. These studies revealed that in most cases, the metal complexes could efficiently inhibit studied enzymes and shown to be highly cytotoxic toward cancer cells both *in-vitro* and *in-vivo*. These findings are relied upon to persuade scientists to look for or develop drugs targeting enzymes as part of efforts to reduce unwanted side effects and improving the activity.

**Scheme 1:**
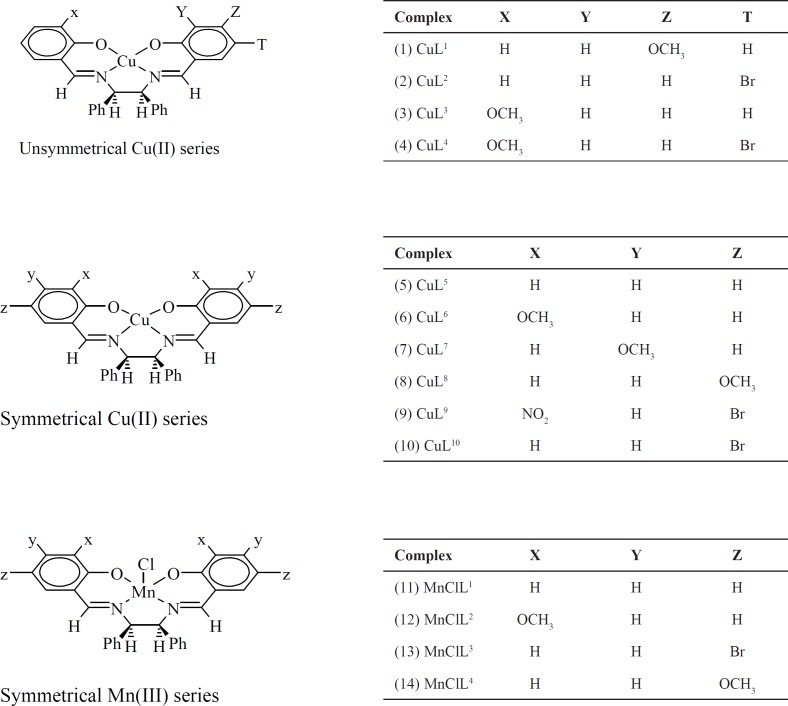
Structures of the complexes used for docking into the active sites of the various receptors

We have previously reported the synthesis and *in-vitro *anticancer activity of several metal complexes, based on square-based pyramidal Mn (III), and square-planar Cu(II), coordinated to salen-type tetradentate ligands (Scheme 1) (17, 18). Considerable biological results (IC_50_ = 14-21 µM in MCF-7 and 10-30 µM in Hep-G2), that some were potent than cis-platin and 5-FU, prompted us to further investigate their pharmacological properties. In an effort to initially understand the binding affinity of these complexes over some anticancer targets, in the present study, we performed *in-silico* docking of our metal complexes with various protein targets and determined their interaction properties including active site binding modes and binding energy. For docking experiments, we have chosen eight protein targets. Among them, two targets belong to histone deacetylases (HDAC7 and HDAC2). One target belongs to cysteine proteases (Cathepsin B (CatB)) and one belongs to kinase family (B-RAF Kinase). The other targets are Topoisomerase II (TopII), Ribonucleotide reductases (RNR), and thymidylate synthase (TS).

Histone deacetylases (HDACs) are a group of metalloenzymes that catalyze the removal of acetyl groups from histone proteins. HDACs have been divided into four structural classes: Class I (HDAC1-3 and 8), class II (HDAC4-9 and 10) and class IV (HDAC11). Class I, II, and IV are Zn^2+^-dependent metalloenzymes, whereas class III metalloenzymes (SI RT1-7) are NAD^+^-dependent enzymes. These enzymes play a major role in variety of biological functions such as differentiation, proliferation, and apoptosis. Consequently, these enzymes are of interest as attractive targets for the development of anticancer drugs (19). In this work, docking studies were carried out on two different classes of HDAC (I, II), using a series of salen-type Schiff base complexes, to compare their potential activity over HDACs. Cathepsin B, a cysteine protease, also plays role at many stages of cancer growth. The observation that there is over expression of CatB in many aggressive tumors has recently led to the suggestion of CatB as a therapeutic target for cancer therapy (20). BRAF gene makes a protein named B-RAF which is one of the three types of RAF kinase family. This protein plays a major role in maintaining the MAP kinase/ERKs activity that promotes cell division, proliferation, differentiation, and secretion. BRAF Kinase represents a special target for anticancer drugs designing (21). Type II topoisomerases (TopII) cut both strands of the DNA helix simultaneously in order to manage DNA tangles and supercoils. TopII is a suitable target for broad range of drugs, including anticancer, antiviral, and antimalarial compounds (22). RNR plays key role in maintaining total rate of DNA formation. Increased its activity has been associated with cancer, and inhibitors of this enzyme are of interest for cancer treatment (14). TS is an essential enzyme in regulating balanced supply of four deoxynucleotides in DNA replication. Therefore, this enzyme could be an important drug target in cancer treatment (15). rHA has important role in the pharmacokinetic availability of the drugs, consequently, affecting their bioavailability properties (23). In this research, we have predicted the inhibitory activity and the possible targets for the studied metal complexes using AutoDockTools. Moreover, the special attempt has been focused on the structure-inhibition relationships.

## Experimental

*Ligand setup*

The 3D structure models of the studied metal complexes were generated by hyperchem 8.0. Geometry optimizations were performed in two steps. At first, the metal complexs were assigned to energy minimization using the ″MM^+^″ force field. Semi-emperical ″PM3″ method was then used to optimize full geometry of the complexes.

*Protein setup*

The crystal structures of the selected targets were extraced from protein data bank and were setup as follows: All water molecules in X-ray structures (except which exists in the active sites), multiple ligands and non protein parts except co-factors, were deleted by ViewerLite4.2 (Table 1). Kollman partial charges and polar hydrogen atoms were assigned to the receptors by AutoDock software.

*Docking calculations*

AutoDock program (using MGL tools 1.5.6) was used for the molecular docking studies. Input files required for docking study were made in AutoDock tools. The grid maps were defined with Autogrid4. For all calculations, we used the grid size with 60 × 60 × 60points and a grid spacing of 0.375 Å. The center of the grid box was set to the center of the ligand in the crystal structure of the related protein. AutoDock4 applied to calculate the binding energy for discovery of the best binding mode. Lamarckian genetic algoritm (LGA), which is available in AutoDock program, was used to perform docking calculations. Fifty indipendent runs for conformational search with maximum number of 2,500,000 energy evaluations per run were carried out. Default values were set for the other operations. The best conformation of the complexes into the active site was identified based on binding affinity score. In fact, the lower the binding energies, the more effective the binding mode. For the validation of AutoDock program, the bound ligands located in the crystal structures were removed from the active site of the related receptors and were re-docked into the active sites. There were good agreement between reproduced binding modes and the original results (RMSD ≤ 2.0 Å), suggesting the high ability of AutoDock4 to generate reliable results.

## Results and Discussion

*Molecular docking*

Although, for successful discovery of new medicinal compounds a combination of theoretical and experimental techniques are required, several computer modeling methods exist to reduce drug development time and cost, which have played critical roles in understanding the enzyme-drug interactions. Docking simulation is an effective tool to predict more potent and specific inhibitors for variety of different protein targets (24). This method is wieldy used to gain insight into the interactions between the enzymes and the metal-based inhibitors (25, 26). Detailed structural information obtained from this method can be employed for guiding a rational drug design. In this study, we used molecular docking technique to evaluate the binding mods between some anticancer targets and the studied Schiff base complexes. Our interest here is to analyze mode of interactions and explore the possible targets for the complexes used. Table 2 shows the calculated binding energies from the docking studies. Tables S1 and S2 (supplementary file) show the van der Waals and hydrogen bonds plus desolvation energy components of calculated docking energies. Electrostatic interactions of each metal complex in the active site of the related targets have been shown in the 4^th^. These results indicate the picture of the predicted potency of the complexes over the investigated targets. Detailed analyses of the docking results are descried in the following sections:

Section 1. Considering the Table 2, it was found that the various targets could be classified into two classes, depending on the binding activities of the metal complexes-targets: The proposed target, as most favorable of the metal complexes from the docking results, was HDAC7 (because of its strongest interactions with all of the complexes). CatB, TS, HDAC2, TopII, RNR, followed by Kinase were predicted as the moderate targets of the complexes. Also, docking results indicated that **2** could be the best inhibitor of HDAC7, TS, and Kinase (Table 2). For CatB, **4** was found as the best inhibitor and **10** was predicted the best inhibitor for RNR. **12** was the best proposed inhibitor for TopII receptor. The proposed best inhibitor of HDAC2 was **8**. A critical look at the docking results showed that the complex **9** has acted as the poor inhibitor in the most cases (Table 2).

Section 2. The results, presented above, showed that the bromo derivatives in both unsymmetrical and symmetrical series of Cu(II) complexes could act as the first potent inhibitor of the most receptors. However, in case of TopII, methoxy derivatives (**1**, **3**, **8**, **12** and **14**), were more potent than the bromo derivatives. Order of activities of the complexes over HDAC7, as the best predicted target, follows as **2 **(5-Br derivative in unsymmetrical series)**, 10 **(5-Br derivative in symmetrical Cu(II) series)**, 13 **(5-Br derivative in Mn(III) series)**, 14 **(5-OCH_3_ derivative in Mn(III) series)**, 8 **(5-OCH_3_ derivative in symmetrical Cu(II) series)**, 4 **(3-OCH_3_-5-Br derivative in unsymmetrical series)**, 11 **(unsubstituted Mn(III))**, 5 **(unsubstituted symmetrical Cu(II))**, 1 **(4-OCH_3_ derivative in unsymmetrical series)**, 12 **(3-OCH_3_ derivative in Mn(III) series)**, 3 **(3-OCH_3_ derivative in unsymmetrical series)**, 7 **(4-OCH_3_ derivative in symmetrical Cu(II) series)**, 6 **(3-OCH_3_ derivative in symmetrical Cu(II) series) and** 9 **(3-NO_2_-5-Br derivative in symmetrical Cu(II) series). Considering this order, it could be demonstrated that the substitutions in 3, 4- positions of salicylaldehyde moieties decreases the binding affinity compared to the parent complex, while substitutions in 5-position of salicylaldehyde moieties leads to an increase in activity. Based on the above discussions, the strength inhibitory of the complexes toward HDAC7 is mainly dependent on the position of the substituents on the Salen ligand. 

**Figure 1:**
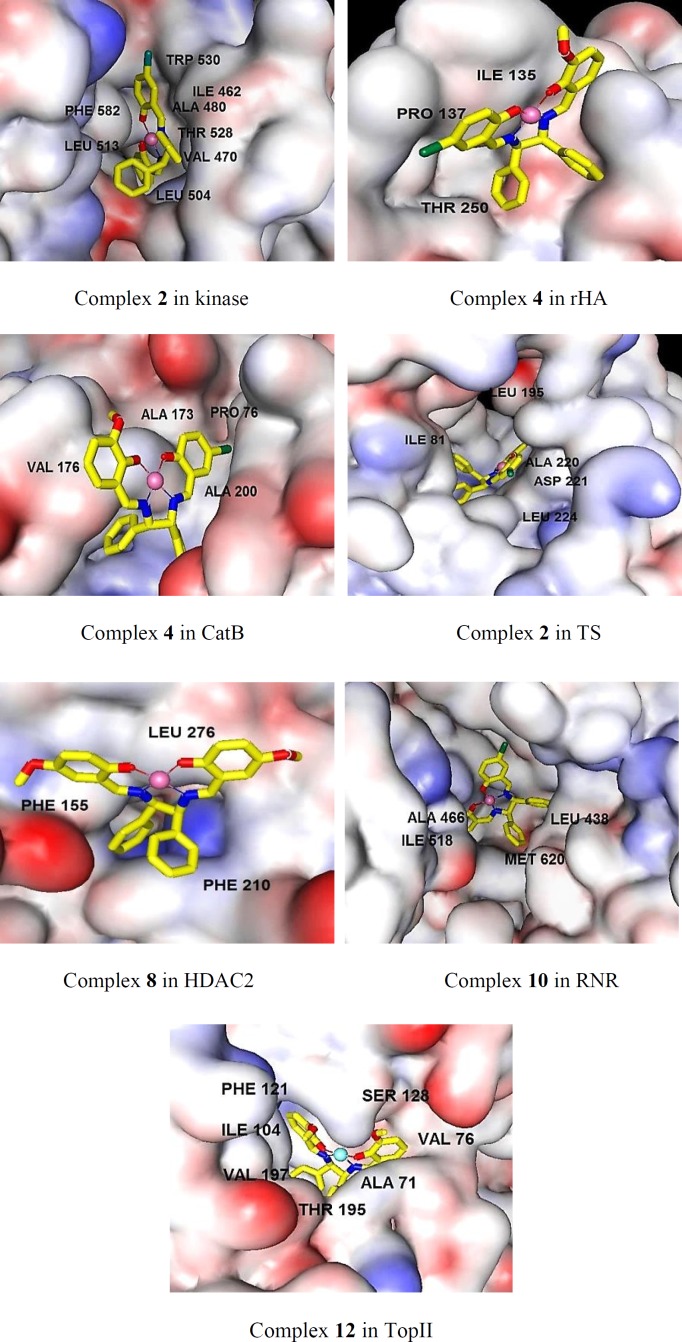
The binding mods of the best inhibitor of the various related targets as predicted by docking method. The main residues involved in hydrophobic interactions were shown

Section 3. The docking study confirmed that the binding modes between the complexes and the receptors were mainly via hydrophobic interactions, aromatic rings and hydrogen bindings. To gain deeper insights into the binding modes of the complexes, the most important interactions for the best inhibitor of each receptor in terms of hydrogen bindings and hydrophobic interactions with the surrounding residues are summarized in Table 3, Figures 1 and S1 (supplementary file). Because of surprisingly high selectivity of all complexes towards HDAC7, the modes of interactions of all complexes with HDAC7 are separately depicted in Section 4. The overall look at Table 3 indicates that inhibitory activities of the complexes are characterized with very few hydrogen bindings and mainly hydrophobic interactions. The features presented in Figure 1 show that in the cases of Kinase, RNR, TS, and TopII, the complexes can penetrate well into the cavity of the receptors presence of higher number of surrounding residues and establishing extensive hydrophobic interactions cause higher stability and lower free energy bindings than the others (Table 3). The orientation of the complexes **2, 10**, and **4** in Kinase, TS, and RNR is in similar manner, in which, the planar arrangement of the salicylaldehyde rings makes the complexes suitable to be inserted into the active site of the receptors to build hydrophobic interactions. Two nonplanar amine rings are oriented towards outside of the active site and contact with residues of the external surface of the binding site. Also, additional hydrophobic interactions of bromo atoms of **2**,** 10**, and** 4 **with surrounding residues gave rise to the potency of these complexes for the related receptors (Figure S1). The planar parts of the complex **4** in CatB and rHA are oriented towards top of the active sites while, the nonplanar amine moieties have penetrated into the active sites of the receptors that lead to weak interactions with the surrounding residues and decrease affinity of **4 **over the receptors. A critical look at the interacting feature of the complex **8 **in HDAC2 indicates that the HDAC2 inhibitor could not penetrate into the channel of the catalytic site of HDAC2 as well as HDAC7, and is characterized with few number of surrounding residues and poor hydrophobic interactions which decrease inhibitor activity of the complex **8** over HDAC2 (Table 3).

**Figure 2:**
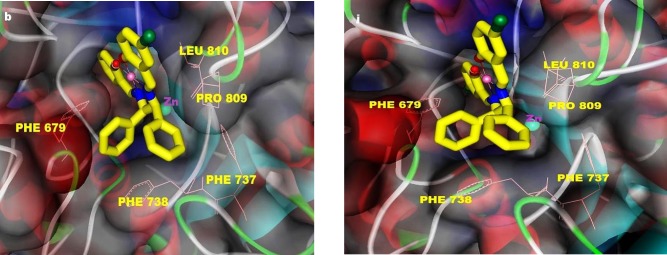
Representative binding mods of the complexes **2 **(left) and **10 **(right) docked into the HDAC7 structure (PDB ID: 3COZ). The main residues involved in hydrophobic interactions were shown

Section 4. In this section, before describing the binding modes and interactions of the complexes with HDAC7, initially, we try to recognize HDAC catalytic pocket. The structural analysis of the active site of HDACs reveals a narrow hydrophobic tunnel-like active site, with a depth of approximately 11Å, that the catalytic zinc ion is located in bottom of the channel. In HDAC7, the walls of the cavity are lined mainly by hydrophobic residues, including PHE738, PRO542, GLY678, PHE679, and LEU810, and Zn^2+^ ion is coordinated by polar residues: ASP707, HIS709, ASP801 (27). In the present work, docking results showed surprisingly high selectivity of all complexes towards HDAC7. Detailed analyses of the binding modes of the complexes are depicted in Table 4, Figures 2, S2 and S3. Figures 2 and S3 show that all complexes could partially penetrate into the hydrophobic channel of the active site of HDAC7 and make often contact with hydrophobic residues of the entrance region of the catalytic site. However, none of the complexes could be close to the Zn binding region where conserved polar residues coordinate to Zn^2+^ ion. Also, docking results revealed a preference for a single location of the complexes into the bonding pocket of HDAC7. The main residues involved in hydrophobic interactions are PHE679, PHE737, PHE738, PRO809, and LEU810 (Table 4). Establishing the hydrogen bindings between one methoxy oxygen of **3** and **4 **with GLY842 were observed (Figures S2c and S2d). Unsymmetrical Cu(II) series are found to penetrate more inside the receptor than the symmetrical Cu(II) derivatives which leads to more fit in to the narrow channel of the active site and make better interactions with surrounding amino acids residues that resulted in an increase in activity respect to symmetrical analogues. The features present in Figures S3a and S2b show that the orientations of **1 **and **2** into the active site of HDAC7 is in similar manner in which, the planar parts of the complexes (salicylaldehyde moieties) insert into a narrow groove of the active site, and make hydrophobic contact with LEU810, PRO809, and PHE679 Whereas, phenyl rings of amine moieties have oriented towards entrance amino acids residues, making hydrophobic interactions with PHE737, PHE738, PHE679, and PRO809 (Figures S2a and S2b). Additionally, bromo atom in **2** is involved in hydrophobic interaction with PRO809 and LEU810 (Figure S2b). Also, the complexes **3** and **4** are oriented alike (Figures S3c and S3d). One of the phenyl rings of amine has contacted with PHE737, PHE738, and PRO809, while the other upper ring could not interact with the receptor. The rings of salycilaldehydes like **1** and **2** could insert into the active site of HDAC7 and establish hydrophobic interactions with LEU810 and PHE679 residues. Furthermore, the methoxy oxygen atoms in **3 **and** 4** are involved in hydrogen bindings with GLY842 (length of hydrogen bond: GLY842: NH…complex **3** = 2.137 Å and GLY842: NH…complex **4 **= 2.43 Ǻ) (Figures S2c and S2d). The complexes **5** and **10** are resemble binding mode of **1** and **2**. As shown in Figures S3e and S3j, the salicylaldehyde rings appear to be oriented towards the channel of the active site and establish hydrophobic contact with LEU810, PHE676, and PRO809 residues. Also, one bromo atom in the case of **10 **(Figure S2j) has formed hydrophobic interactions with mentioned amino acids residues. In the same way as **1** and **2**, the two amine rings contact with following hydrophobic residues, located in the external surface of the catalytic site: PHE679, PHE737, PHE738 and PRO809. In the cases of **8** and **9**, in which, the complexes have an inverted orientation with respect to the other Cu(II) complexes (Figures S3h and S3i), amine moieties are located into the channel of the active site and made some hydrophobic interactions with LEU810, PHE679, and PHE738. The upper phenyl ring in both cases, is oriented in such a way that a π-π stacking interaction with HIS843 additional to hydrophobic interactions is established. The planar parts of the complexes lie at the top of the active site in a large region but, have established less number of hydrophobic interactions with amino acids residues. In complex **8**, one of the salicylaldehyde rings could interact with PRO809 and PHE737 while, no interaction between the other ring and the residues is observed. Furthermore, in complex **9**, the salicylaldehyde moieties (except one bromo atom which could contact with PHE737) do not seem to interact with the receptor. Thus, the weaker activity of the complex **9** might be ascribed to the nonproductive orientation of the salicylaldehyde moieties. In the complex **7**, as shown in Figure S3g, the planar part inserts partially into the active site groove enabling some hydrophobic interactions with PHE738, and PHE679 together with establishing a π-π stacking interaction between outside ring of salicylaldehyde and PHE738 residue. The two amine rings orient to a region composed mainly by the following residues; PHE679, LEU810, and PRO809. The complex **6** presents a very different feature from the other complexes (Figure S3f). An interesting feature is the position of the salicylaldehyde moieties that lie completely at top of the active site and contact with THR625 residue that has not been observed in the other cases, in addition to PHE737, PHE738, and PRO809. The two phenyl rings of amine moiety appear to be oriented into the active site such that a π-π stacking interaction with HIS709 additional to PHE679, and LEU810 can occur. As illustrated in Figures S3k-S3n, the Mn(III) derivatives have very similar binding modes (except parent complex). The two amine rings could penetrate into the active site and establish a π-π stacking interaction with HIS843 additional to hydrophobic interactions with PHE679, PHE738, and LEU810 residues. The salicylaldehyde moieties are located in the same way that was observed in **8**. The parent complex of Mn(III) series has presented different binding mode with respect to the other Mn(III) derivatives. The mode of interaction of complex **11 **(Figure S3k) is similar to that of complex **5** in which, the complex **11** is located in the same binding pocket as the complex **5**. It is interesting to note that the presence of axial chlorine atom in Mn(III) derivatives does not appear to have a significant effect on HDAC7 binding. According to the overall interacting features of the docked complexes with HDAC7, we conclude the following remarks: i) Metal-Salen type Schiff base complexes can bind effectively to HDAC7 by hydrophobic interactions. Planar groups that coordinate the central metal can play a major stabilizing role. ii) The bromo complexes are more effective than the other derivatives because the electronegativity of the bromo atoms would be expected to promote stacking interactions for the aromatic rings of the salicylaldehyde moiety. In addition, the presence of bromo atoms in hydrophobic interactions with surrounding residues may increase stability of the complexes into the active site. iii) The position of the OCH3 substitute is important. According to docking data, 3, 4-OCH_3_ derivatives of Cu(II) complexes (3-OCH_3_ in Mn(III) derivatives) showed the lowest activity on HDAC7 with respect to 5-OCH_3_ derivatives (Section 2). The reason might be the strict effects of the methoxy groups in 3 and 4 positions of the complexes especially Cu(II) derivatives, which prevent appropriate orientation of these complexes within the active site and cause a drop in activity. iv) The affinity of the Mn(III) derivatives for the HDAC7 is very close together (binding data vary between -8.1and -8.94 kcal.mol^-1^) with respect to corresponding Cu(II) analogues, therefore, it seems that the nature and position of the substituents on salicylaldehyde rings play a minor role in defining the activity of these complexes, although the bromo-substituted one induce higher activity over HDAC7 than the other derivatives.

**Table 1:** Protein targets and pdbs selected for the study

**Target**	**Selected PDB (resolution)**	**Co-crystalized ligand**	**Chains**	**Selected chain, (s)**
HDAC7	3COZ (2.1 Ǻ)	Octanedioic acid hydroxylamine phenylamide	A, B, C	B
HDAC2	3MAX (2.05 Ǻ)	N-(4-aminobiphenyl-3-yl)benzamide	A, B, C	A
CatB	1CSB (2 Ǻ)	N-[(3R)-4-ethoxy-3-hydroxy-4- oxobutanoyl]- L-isoleucyl-L-proline CA030	A, B	A, B
B-RAF kinase	3Q4C (3.2 Ǻ)	C25 H16 Cl N3 O4 Ru	A, B	A
TopII	1QZR (1.9 Ǻ)	(S)-4,4'-(1-methyl-1,2-ethanediyl)bis-2,6- piperazinedione	A, B	B
RNR	4R1R (3.2 Ǻ)	guanosine-5'-diphosphate	A, B, C	C
TS	2G8D (2.4 Ǻ)	2'-deoxyuridine 5'-monodiphosphate	A	A
rHA	1BMO (3.1 Ǻ)	n-acetyl-d-glucosamine	A, B	A

**Table 2:** The minimum binging energy of the docked structures (kcal mol-1).

**Complex**	**CatB**	**HDAC7**	**HDAC2**	**Kinase**	**rHA**	**RNR**	**TopII**	**TS**
1	-6.28	-8.14	-6.50	-6.77	-6.28	-7.59	-7.33	-6.87
2	-6.45	-9.60	-6.42	-8.19	-7.09	-7.38	-7.07	-8.31
3	-6.41	-7.89	-6.10	-6.92	-7.15	-7.05	-7.13	-6.78
4	-7.29	-8.33	-6.20	-7.87	-7.32	-7.07	-7.11	-6.89
5	-6.19	-8.20	-6.01	-7.67	-6.34	-6.98	-6.03	-7.08
6	-6.07	-7.50	-6.02	-7.40	-6.39	-7.00	-5.95	-6.56
7	-6.50	-7.52	-5.78	-6.76	-6.91	-7.32	-6.68	-6.61
8	-6.95	-8.55	-7.09	-7.67	-7.25	-7.61	-7.85	-7.25
9	-6.14	-7.05	-4.80	-6.50	-5.81	-5.67	-5.65	-5.70
10	-6.76	-9.50	-6.06	-8.12	-6.79	-8.25	-7.07	-7.85
11	-7.03	-8.31	-6.31	-6.13	-6.14	-7.18	-6.11	-6.53
12	-6.84	-8.10	-6.51	-6.58	-6.11	-6.50	-7.91	-7.18
13	-6.55	-8.94	-6.00	-7.69	-6.68	-7.65	-6.92	-7.17
14	-6.38	-8.87	-6.01	-8.13	-6.54	-7.79	-7.26	-7.40

**Table 3:** The interactions of the binding site residues with the best inhibitor of the related receptor

**Receptor-complex**	**Binding energy**	**Surrounding residues**
**CatB **(4)	-7.29	GLY198B*, *GLU122A, GLY27A, GLN23A, CYS29A, GLY74B, TRP30A, **VAL176B**, GLY197B, HIS199B, **MET196B**, **ALA200B**, **PRO76B**, **ALA173B**
**HDAC2 **(8)	-7.09	HIS33, PRO34, **LEU276**, **PHE155**, ASP104, GLY154, **PHE210**, HIS183,
**Kinase **(2)	-8.19	CYS531, **TRP530**, **LEU513**, **PHE582**, PHE594, ASN580, ASP593, LYS482, GLY463, **ILE462**, **VAL470**, SER464, PHE467, **THR528**, **ALA480**, **LEU504**,
**rHA **(4)	-7.32	ASP127, LYS133, SER96, ASN97, ASN99, ASP98, **THR250**, TYR134, **ILE135**, **PRO137**
**RNR **(10)	-8.25	SER625, LEU207, SER206, **ALA466**, SER465, LEU464, PRO621, **THR209, **SER224, LY253, CYS225, ASN437, **LEU438**, CYS439, **ILE518**, **MET620**
**TopII **(12)	-7.91	**ILE104**, **PHE121**, ARG77, ASP63, **THR138**, ASN129, **SER128**, ASP73, ASN99, **THR195**, ASN70, SER127, **ALA71**, **VAL197**, **ILE67**, **VAL76**
**TS **(2)	-8.31	GLU60, **ILE81**, CYS196, ASN229, **PHE228**, **LEU224**, GLY225, **ASP221**, **ALA220**, HIS250, **TRP82**, **LEU195**, **VAL314**

The first column shows the receptor and the number of its best active complex. The last column shows the surrounding residues of the docked complexes. Residues, which form hydrophobic interaction with the complexes are highlighted in bold and residues, which form hydrogen bonds are written in italic.

**Table 4:** The interactions of the binding site residues of HDAC7 with the complexes

**Complex**	**Binding energy**	**Surrounding residues**
1	-8.14	**PHE679**, **PHE737**, **PHE738**, **PRO809**, **LEU810**, ARG547, HIS843, ASP626, HIS709, GLY842, GLY841
2	-9.6	**PHE679**, **PHE737**, **PHE738**, **PRO809**, **LEU810**, ARG547, HIS843, ASP626, HIS709, GLY842, GLY841, PRO542, GLY673, GLU543
3	-7.89	**PHE679**, **PHE737**, **PHE738**, **PRO809**, **LEU810**, *GLY842, *HIS843, ASP626, HIS709, GLY842, GLY841, PRO542, PRO609, ASP801
4	-8.33	**PHE679**, **PHE737**, **PHE738**, **PRO809**, **LEU810**, *GLY842*, HIS843, ASP626, HIS709, GLY842, GLY841, PRO542, PRO609, ASP801
5	-8.2	**PHE679**, **PHE737**, **PHE738**, **PRO809**, **LEU810**, APG547, PRO542, GLU543, GLY676, HIS709, HIS843, GLY841
6	-7.5	**PHE679**, **PHE737**, **PHE738**, **PRO809**, **LEU810**, **HIS709**, **THR625**, ASP624, ASP626, GLY678
7	-7.52	**PHE679**, **PHE738**, **PRO809**, **LEU810**, **PRO542**, GLY678, ASP624, ASP626, HIS709, HIS670, HIS669, GLY841, ASP801
8	-8.55	**PHE679**, **PHE737**, **PHE738**, **PRO809**, **LEU810**, **HIS843**, APG540, PRO542, HIS709, GLY841, ASN739
9	-7.05	**PHE679**, **PHE737**, **PHE738**, **LEU810**, **HIS843**, ASN736, GLY678, GLY842, PRO542, ASP801
10	-9.5	**PHE679**, **PHE737**, **PHE738**, **PRO809**, **LEU810**, HIS709, ASP626, PRO667, GLU543, PRO542, ARG547, GLY841, GLU840
11	-8.31	**PHE679**, **PHE737**, **PHE738**, **PRO809**, **LEU810**, HIS709, ASP626, GLY842, GLY841, HIS843
12	-8.06	**PHE679**, **PHE737**, **PHE738**, **PRO809**, **LEU810**, **HIS843**, ASP626, HIS709, GLY842, GLY678
13	-8.94	**PHE679**, **PHE737**, **PHE738**, **PRO809**, **LEU810**, **HIS843**, HIS709, PRO542, GLY842, GLY678
14	-8.87	**PHE679**, **PHE737**, **PHE738**, **PRO809**, **LEU810**, **HIS843**, HIS709, ASN736, GLY842, GLY678, PRO542, ARG540

The last column shows the surrounding residues of the docked complexes. Residues, which form hydrophobic and π-π stacking

interactions with the complexes are highlighted in bold and residues, which form hydrogen bonds are written in italic.

## Conclusion

Through this research, docking strategy approach has allowed us to predict inhibitory activity and the possible protein targets for some Salen-type Schiff base transition metal complexes. This study revealed that the most of the studied complexes are able to interact with the active pocket of the investigated targets and the mainly hydrophobic contacts contribute to the stability of the complexes. Understanding the exact nature of the interactions is key for controlling drug specificity and reducing its side effects. The HDACs inhibition results are highly encouraging. An overlay of the binding modes of the complexes shows that all complexes have preferred to the best bind to HDAC7, which is an indication of their specificity towards HDAC7. While, HDAC2 is predicted to be poor target. Up to now, many type of organic HDACs inhibitors are identified, and several effective metal-based analogous are currently under development (28, 29). While, Such HDACs inhibitors often exhibit chelate interactions with the catalytic Zn^2+^ ion, the complexes used in this study are rendering the interactions with the mouth of the catalytic site cleft. Given that the structural elements in the entrance of the active site are different among the various HDACs, the compounds, able interact with this region of the protein provide new approaches toward isozyme specificity.

## References

[1] Rosenberg B (1978). Platinum Complexes for the Treatment of Cancer. Interdiscip. Sci. Rev.

[2] Leite SM, Lima LM, Gama S, Mendes F, Orio M, Bento I, Paulo A, Delgado R, Iranzo O (2016). Copper(II) complexes of phenanthroline and histidine containing ligands: synthesis, characterization and evaluation of their DNA cleavage and cytotoxic activity. Inorg. Chem.

[3] Mjos MD, Orvig C (2014). Metallodrugs in medicinal inorganic chemistry. Chem. Rev.

[4] Gasser G, Ott I, Metzler-Nolte N (2011). Organometallic anticancer compounds. J. Med. Chem.

[5] Manikandan R, Viswnathamurthi P (2012). Coordination behavior of ligand based on NNS and NNO donors with ruthenium (III) complexes and their catalytic and DNA interaction studies Spectrochim. Acta A Mol. Biomol. Spectrosc.

[6] Feng L, Geisselbrecht Y, Blanck S, Wilbuer A, Atilla-Gokcumen GE, Filippakopoulos G, Celik MA, Harms K, Maksimoska J, Marmorstein R, Frenking G, Knapp S, Essen LO, Meggers E (2011). Structurally sophisticated octahedral metal complexes as highly selective protein kinase inhibitors. J. Am. Chem. Soc.

[7] Yang M, Pickard AJ, Qiao X, Gueble MJ, Day CS, Kucera GL, Bierbach U (2015). Synthesis, reactivity, and biological activity of gold(I) complexes modified with thiourea-functionalized tyrosine kinase inhibitors. Inorg. Chem.

[8] Spencer J, Amin J, Wang MH, Packham G, Alwi SS, Tizzard GJ, Coles SJ, Paranal RM, Bradner JE, Heightman TD (2011). Synthesis and biological evaluation of JAHAs: ferrocene-based histone deacetylase inhibitors. ACS Med. Chem. Lett.

[9] Kasparkova J, Kostrhunova H, Novakova O, Křikavová R, Vančo J, Trávníček Z, Brabec V (2015). A photoactivatable platinum(IV) complex targeting genomic DNA and histone deacetylases. Angew. Chem. Int. Ed. Engl.

[10] Ruiz J, Vicente C, Haro C, Bautista D (2013). Novel bis-C,N-cyclometalated iridium(III) thiosemicarbazide antitumor complexes: interactions with human serum albumin and DNA, and inhibition of cathepsin B. Inorg. Chem.

[11] Rojas S, Carmona FJ, Barea E and Maldonado CR (2017). Inorganic mesoporous silicas as vehicles of two novel anthracene-based ruthenium metalloarenes. J. Inorg. Biochem.

[12] Fillipe V, Rochaa FV, Barraa CV, Garridoc SS, Manented FA, Carlosd IZ, Ellenae J, Fuentesf AS, Gautierg A, Morelh L, Mauroa AE, Netto AV (2016). Cationic Pd(II) complexes acting as topoisomerase II inhibitors: synthesis, characterization, DNA interaction and cytotoxicity. J. Inorg. Biochem.

[13] Sandhausa S, Taylorb R, Edwardsb T, Huddlestonb A, Wootenb Y, Venkatramanc R, Weberd RT, González-Sarríase A, Martinf PM, Caglef P, Tse-Dinha YC, Beebeh S, Seerame N, Holderi AA (2016). A novel copper(II) complex identified as a potent drug against colorectal and breast cancer cells and as a poison inhibitor for human topoisomerase IIα. Inorg. Chem. Commun.

[14] Zaltariov MF, Hammerstad M, Arabshahi HJ, Jovanović K, Richter KW, Cazacu M, Shova S, Balan M, Andersen NH, RadulovićS, Reynisson JH, Andersson KK, Arion VB (2017). New Iminodiacetate−thiosemicarbazone hybrids and their copper(II) complexes are potential Ribonucleotide reductase R2 inhibitors with high antiproliferative activity. Inorg. Chem.

[15] Pelà M, Saxena P, Luciani R, Santucci M, Ferrari S, Marverti G, Marraccini C, Martello A, Pirondi S, Genovese F, Salvadori S, D’Arca D, Ponterini G, Costi MP, Guerrini R (2014). Optimization of peptides that target human thymidylate synthase inhibit on ovarian cancer cell growth. J. Med. Chem.

[16] Tavares TT, Azevedo GC, Garcia A, Carpanez AG, Lewer PM, Paschoal D, Müller BL, Dos Santos HF, Matos RC, Silva H, Grazul RM, Soares Fontes AP (2017). Gold(I) complexes with aryl-thiosemicarbazones: molecular modeling, synthesis, cytotoxicity and TrxR inhibition. Polyhedron.

[17] Damercheli M, Dayyani D, Behzad M, Mehravi B, Shafiee Ardestani M (2015). New Salen-type manganese(III) Schiff base complexes derived from meso-1,2-diphenyl-1,2-ethylenediamine: in-vitro anticancer activity, mechanism of action, and molecular docking studies. J. Coord. Chem..

[18] Behzad M, Seifikar Ghomi L, Damercheli M, Mehravi B, Shafiee Ardestani M, Jahromi HS, Abbasi Z (2016). Crystal structures and in-vitro anticancer studies on new unsymmetrical copper(II) Schiff base complexes derived from meso-1,2-diphenyl-1,2-ethylenediamine: a comparison with related symmetrical ones. J. Coord. Chem.

[19] Micelli D, Rastelli G (2015). Histone deacetylases: structural determinants of inhibitor selectivity. Drug Discov. Today.

[20] Aggarwal N, Sloane BF (2014). Cathepsin B: multiple roles in cancer. Proteomics Clin. Appl..

[21] Xie P, Streu C, Qin J, Bregman H, Pagano N, Meggers E, Marmorstein R (2009). The crystal structure of BRAF in complex with an organoruthenium inhibitor reveals a mechanism for inhibition of an active form of BRAF kinase. Biochem.

[22] Peng-Hui L, Ping Z, Shuo-Bin C, Pei-Fen Y, Yan-Wen M, Jia-Heng T, Tian-Miao O, Shi-Liang H, Ding L, Lian-Quan G, Zhi-Shu H (2016). Synthesis and mechanism studies of 1,3-benzoazolyl substituted pyrrolo[2,3-b]pyrazine derivatives as non-intercalative topoisomerase II catalytic inhibitors. J. Med. Chem.

[23] Hu W, Luo Q, Ma X, Wu K, Liu J, Chen Y, Xiong S, Wang J, Sadler PJ, Wang F (2009). Arene control over thiolate to sulfinate oxidation in albumin by organometallic ruthenium anticancer complexes. Chem. Eur. J.

[24] Farrokhnia M, Karim Mahnam K (2017). Molecular dynamics and docking investigations of several zoanthamine- type marine alkaloids as matrix metaloproteinase-1 inhibitors. Iran. J. Pharm. Res.

[25] Pravina N, Devarajib V, Ramana N (2015). Targeting protein kinase and DNA molecules by diimine–phthalate complexes in antiproliferative activity. Int. J. Boil. Macromol..

[26] Adeniyi AA, Ajibade PA (2012). Inhibitory activities and possible anticancer targets of Ru(II)-based complexes using computational docking method. J. Mol. Graph. Model..

[27] Schuetz A, Min J, Allali-Hassani A, Schapira A, Shuen M, Loppnau M, Mazitschek P, Kwiatkowski R, Lewis NP (2008). Human HDAC7 harbors a class IIa histone deacetylase-specific zinc binding motif and cryptic deacetylase activity. J. Biol. Chem.

[28] Leonidova A, Mari C, Aebersold C, Gasser G (2016). Selective Photorelease of an organometallic-containing enzyme inhibitor. Organometallics.

[29] Cross JM, Blower TR, Gallagher N, Gill JH, Rockley KL, Walton JW (2016). Anticancer RuII and RhIII piano-stool complexes that are histone deacetylase inhibitors. ChemPlusChem.

